# A Precise Focusing Simulation Platform for Transcranial Acoustoelectric Brain Imaging

**DOI:** 10.3390/s26092715

**Published:** 2026-04-28

**Authors:** Jiande Guo, Juan Huang, Xizi Song, Chenghao Hu, Xiuyun Liu, Dong Ming

**Affiliations:** 1 State Key Laboratory of Advanced Medical Materials and Medical Devices, Academy of Medical Engineering and Translational Medicine, Medical School, Tianjin University, Tianjin 300072, China; guojiande@tju.edu.cn (J.G.); richardming@tju.edu.cn (D.M.); 2 Institute of Acoustics, Chinese Academy of Sciences, Beijing 100190, China; huangjuan@mail.ioa.ac.cn; 3 State Key Laboratory of Acoustics and Marine Information, Institute of Acoustics, Chinese Academy of Sciences, Beijing 100190, China; huchenghao@mail.ioa.ac.cn

**Keywords:** acoustoelectric brain imaging, transcranial focused ultrasound, precise focusing simulation platform, skull

## Abstract

Transcranial acoustoelectric brain imaging (tABI) is a potential brain activity imaging technique with high spatiotemporal resolution. Precise focus of transcranial ultrasound is critical for realizing millimeter-level spatial resolution in tABI. In this study, a precise focusing simulation platform is proposed by constructing a high-precision 3D skull model from Bama pig computed tomography data and a mathematical model equation by considering the skull’s heterogeneous properties. Then, the delay parameters derived from the simulation platform improve the precision of transcranial ultrasound focusing, enabling precise localization of brain activation sources with tABI. Phantom experimental results demonstrate that the transcranial ultrasound field is precisely focused at the target with a 0.20 mm deviation when delay parameters are obtained from the proposed simulation platform, whereas it exhibits divergence when using delay parameters derived from pure water or homogeneous skull models. Furthermore, using the proposed simulation platform, tABI can accurately identify intracranial electrical signals of distinct frequencies and precisely locate the corresponding activation sources with a spatial deviation of 0.50 mm. These results demonstrate that the proposed simulation platform is a powerful tool for the precise focusing of tABI.

## 1. Introduction

Neuroimaging plays an irreplaceable clinical and research role in areas such as epilepsy focus localization, sleep stage analysis, cognitive function assessment, and early diagnosis of brain diseases [1,2,3]. However, current neural signal acquisition technologies still face significant challenges. Electroencephalography (EEG) is affected by signal attenuation caused by the scalp, skull, and other tissues, making it difficult to overcome noise interference at the hundred microvolt level and the limitations of spatial resolution at the centimeter scale [4,5]. Electrocorticography (ECoG) recording relies on invasive electrode implantation, which presents issues such as poor biocompatibility, insufficient long-term recording stability, and postoperative complication risks. Optical brain activity recording techniques, such as functional near-infrared spectroscopy (fNIRS) and cortical optical imaging, can provide complementary metabolic information, but their temporal resolution and synchronization with electrophysiological signals require further improvement [6]. The emerging technology of transcranial acoustoelectric brain imaging (tABI) is a neuroimaging technique that combines the millimeter-scale spatial resolution of ultrasound with the millisecond-scale temporal resolution of EEG signals [7,8,9,10,11]. Based on a high spatiotemporal resolution and non-invasive nature, tABI holds promise as a functional brain imaging technology for exploring the mechanisms of the brain’s neural system [12,13,14,15,16]. It has garnered significant attention in the fields of brain electrical signal detecting and brain activation source distribution. It is important to note that tABI requires higher spatial precision of transcranial ultrasound focusing, making accurate transcranial ultrasound focusing one of the key challenges that tABI technology must address [17,18,19]. Ultrasounds have been used for diagnostics and treatment for many years, but it is only in the past two decades that they have been explored as diagnostic and research tools for the brain in the field of neuroscience [20,21]. Therefore, achieving precise focusing of transcranial ultrasound is a critical challenge in both clinical and research settings [22]. Due to factors such as the complex structure and heterogeneity of the skull, the ultrasound beam often cannot be focused on the intended focal spot [23,24,25,26]. By precisely compensating for the phase errors caused by the skull’s attenuation of the ultrasound, phase discrepancies during propagation can be corrected. To achieve phase compensation, the most effective method is the implantation of a hydrophone, which involves placing a hydrophone at the target point inside the skull to receive signals emitted by the phased array outside the skull. The hydrophone captures the phase delays of the signals arriving at the target point from each element of the array [27]. Then, corresponding delay parameters are applied to each channel of the phased array to achieve phase compensation. This method can yield accurate phase compensation; however, the process of implanting a hydrophone in the brain is invasive and may potentially cause irreversible damage to the brain [28].

Therefore, resolving the challenge of precise transcranial ultrasound focusing is essential for improving the spatial resolution of tABI. In this study, we propose a numerical simulation platform for precise transcranial ultrasound focusing, which combines a high-precision 3D skull model constructed from CT data with a mathematical model that fully accounts for skull structural heterogeneity and density. The platform generates the phased array delay parameters required for accurate transcranial focusing using time-reversal phase control. The effectiveness and focusing accuracy of the platform are validated through both numerical simulations and phantom experiments. Using this platform, tABI achieves precise detection and localization of intracranial activation sources.

## 2. Theory

### 2.1. Precise Focusing Simulation Platform

#### 2.1.1. Skull Structural Model

**Figure 1:**
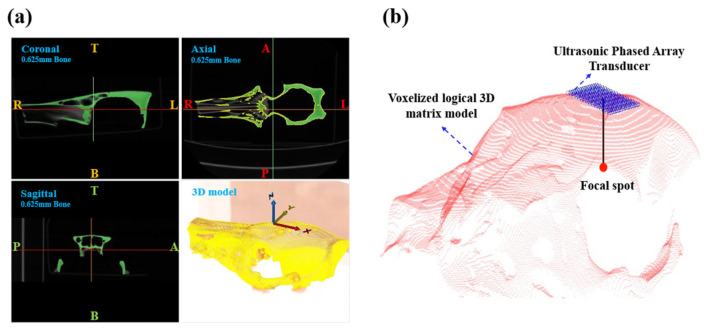
(**a**) The CT image of pig skull. (**b**) The 3D numerical skull model is created using the CT data of the pig skull.

**Figure 2:**
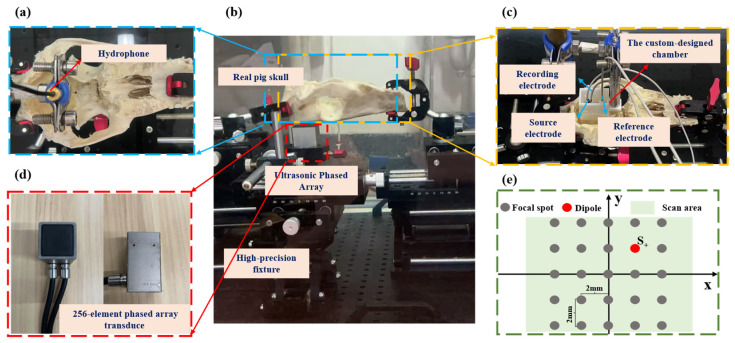
The phantom experimental setup diagram. (**a**) The scene diagram showing the hydrophone measuring the transcranial ultrasound field. (**b**) The ultrasonic phased array, skull and fixture placement diagram. (**c**) tABI experimental setup diagram. (**d**) Customized 256-element ultrasonic phased array appearance diagram. (**e**) The schematic of the ultrasound scanning plane.

As shown in Figure 1, the 3D skull model is created using the computerized tomography (CT) data of the pig skull. As fresh human skulls are extremely difficult to obtain, porcine skulls serve as an ideal surrogate for investigating the transcranial ultrasound characteristics. Additionally, porcine skulls’ cortical bone structure, thickness, and acoustic properties are comparable to those of human skulls, which makes them ideal for transcranial ultrasound studies [29,30]. The skull model is imported into MATLAB R2020a (MathWorks Inc.), where it is converted into a voxelized logical 3D matrix with isotropic spatial discretization (dx = 0.4766 mm, dy = 0.4766 mm, dz = 0.6250 mm) matching that of the simulation domain in Figure 1b [31]. In the simulation model, a 256-element planar phased array with array element spacing of 1.5 mm is constructed, identical to the customized phased array we designed. As shown in Figure 2d, the side length of the customized 256-element planar phased array is 34 mm. For numerical simulation, the transducer is positioned directly above the posterior half of the sagittal suture. The phased array surface is located approximately 3 mm away from the skull surface along the propagation axis. The skull thickness is 7 mm along the ultrasound propagation path. The simulation domain comprises a skull immersed in pure water, with its internal pores saturated by the pure water medium.

The CT images provide the spatial distribution of Hounsfield units (HU), which quantitatively reflect the linear X-ray attenuation coefficient of skull tissues. Due to the porous nature of the skull, its X-ray attenuation varies with porosity, resulting in an approximately linear relationship between the HU value H and porosity ϕ. Accordingly, the skull porosity ϕ can be directly calculated from the HU value H of each pixel point in the CT image by

(1) $$\phi =1-\frac{H}{1000}$$

Further, these acoustic parameters—density ρ, sound velocity v and sound absorption coefficient α—can be calculated through the porosity ϕ of the skull [32,33,34,35]:

(2) $$\left\{\begin{array}{c} \rho =\phi \rho_{w}+(1-\phi )\rho_{b}\\ c=c_{w}+(1-\phi )(c_{b}-c_{w})\\ \alpha =\phi^{0.5}(\alpha_{b}-\alpha_{w}) \end{array}\right.$$

where $\rho_{w}$ and $c_{w}$ are the density and sound speed of liquid water, respectively. $\rho_{b}$ and $c_{b}$ are density and sound speed of the skull, respectively. α is the sound absorption coefficient, and $\alpha_{b}$ and $\alpha_{w}$ are the maximum and the minimum sound absorption coefficients with the skull, respectively. In the calculations, the acoustic parameters for pure water are set as $\rho_{w}=997\;{\mathrm{kg}/m}^{3}$ [36] and $c_{w}=1500\;m/s$ [37], while the acoustic parameters for the skull are set as $\rho_{b}=1912\;{\mathrm{kg}/m}^{3}$ [38] and $c_{b}=2900\;m/s$ [37]. In the skull model, the skull is considered as a porous medium consisting of the bone medium and water within the pores. Consequently, the medium with the maximum absorption coefficient is the bone medium, for which the absorption coefficient is set to $\alpha_{b}=13\;\mathrm{dB}/\mathrm{cm}/\mathrm{MHz}$ [36], while the medium with the minimum absorption coefficient is pure water, with an absorption coefficient set to $\alpha_{w}=1\;\mathrm{dB}/\mathrm{cm}/\mathrm{MHz}$.

#### 2.1.2. Calculation of the Transcranial Ultrasound Field

The k-Wave toolbox-based calculation method is employed as the benchmark for validation. In this study, the model developed is likewise implemented in the k-Wave toolbox, utilizing a pseudo-spectral approach and first-order fluid coupling equation [39,40,41]. Firstly, the characteristics of the ultrasound field can be represented by the sound pressure P, the particle velocity vector $\vec{v}$, and the density variation ρ within the medium, while the sound waves propagate in the uniform ideal medium. Each acoustic variable can be regarded as a first-order infinitesimal at small amplitude sound waves. Based on Newton’s Second Law of Motion, law of conservation of mass, and the equation of state for an ideal medium, the first-order partial differential equations can be derived to describe

(3) $$\frac{\partial v}{\partial t}=-\frac{1}{\rho_{0}}\nabla P$$

(4) $$\frac{\partial \rho }{\partial t}=-\rho_{0}\nabla \cdot \vec{v}$$

(5) $$P=c_{p0}^{2}\;\rho_{0}$$

where $\rho_{0}$ is the static density of the medium without sound disturbance, $c_{p0}$ is the propagation speed of sound waves in the medium, ∇ is the Nabla operator, ∇A is the gradient of the scalar A, and $\nabla \cdot \vec{A}$ is the divergence of the vector $\vec{A}$.

By differentiating Equation (5) with respect to time t and substituting it into Equation (4), Equation (6) can be obtained as follows:

(6) $$\frac{\partial P}{\partial t}=-\rho_{0}c_{p0}^{2}\nabla \cdot \vec{v}$$

Then, Equation (7) can be obtained by differentiating Equation (6) with respect to time t and substituting it into Equation (3). Equation (7) is the wave equation of sound wave propagation in homogeneous ideal fluid medium, also known as linear wave equation.

(7) $$\nabla^{2}P-\frac{1}{c_{p0}^{2}}\frac{\partial^{2}P}{\partial t^{2}}=0$$

However, sound waves usually lose energy during propagation. The lost energy is related to frequency and is expressed by the sound absorption coefficient:

(8) $$\alpha_{p}=\alpha_{p0}f^{\beta }$$

where α is the power-law sound absorption coefficient with $dB\cdot {MHz}^{-\beta }m^{-1}$, and β is the power-law sound absorption index.

Considering the inhomogeneity of the skull and the sound absorption properties, the static density partial derivative terms and the sound absorption operator L are added to the wave equations of Equations (4) and (5). The operator L is a differential operator that represents the absorption and dispersion of sound waves, and can be expressed using the Nabla operator and a power-law absorption coefficient.

(9) $$L=\tau \frac{\partial }{\partial t}(-\nabla^{2}{)}^{\frac{\beta }{2}-1}+\eta (-\nabla^{2}{)}^{\frac{\beta +1}{2}-1}$$

(10) $$\tau =-2\alpha_{p0}c_{p0}^{\beta -1}$$

(11) $$\eta =2\alpha_{p0}c_{p0}^{\beta }tan(\frac{\pi \beta }{2})$$

where τ and η denote the absorption and dispersion scaling factors, respectively. These two parameters govern the frequency dependence of ultrasonic attenuation and waveform dispersion. Since dispersion is typically neglected in transcranial ultrasound simulations [42], β is assigned a value close to 1 to minimize waveform dispersion; in this study, β is set to 1.05 [43].

Then, Equations (4) and (5) can be rewritten as follows:

(12) $$\frac{\partial \rho }{\partial t}=-\rho_{0}\nabla \cdot \vec{v}-\vec{v}\cdot \nabla \rho_{0}$$

(13) $$P=c_{p0}^{2}\;(\rho +\vec{u}\cdot \nabla \rho_{0}-L\rho )$$

where $\vec{u}$ represents the displacement of the acoustic volume element.

As described above, the density ρ, sound velocity v and sound absorption coefficient α of the skull need to be determined when solving the wave equation using the pseudo-spectral method. However, the parameters ρ, v and α are heterogeneous because of the complex internal structure of the skull, and these differences greatly affect the attenuation and phase distortion of transcranial ultrasound propagation. In this study, these acoustic parameters are determined from the HU value H derived from CT imaging, as demonstrated in Equations (1) and (2).

Shear waves are not considered in the simulation calculations of this study. Shear waves can only be effectively excited when the incident angle exceeds 35°, and their excitation amplitude is greater than that of longitudinal waves when the incident angle is larger than 40°. In this study, the ultrasonic phased array is placed directly above the skull, which results in an incident angle of 34.22° for the furthest element in the 256-element linear array used. Therefore, the acoustic waves in the skull are predominantly dominated by longitudinal waves during the focusing process. Meanwhile, nonlinear settings in the intracranial model have no effect on the calculation of time delays or the time-reversal phase control effect [44], so nonlinear terms are excluded from the computations.

Finally, based on the time-reversal phase control method, a virtual point pressure source is placed at the expected focal spot within the intracranial cavity. The process of a virtual point source emitting the ultrasonic and propagating outward is simulated. Subsequently, the constructed ultrasonic phased array receives the ultrasound signals transmitted through the skull from the virtual point source. The time-phase delay signals for the phased array are optimized using the Hilbert transform, and the phased delay parameters are established. Then, a second transcranial simulation is performed, and the transcranial ultrasound field distribution is mapped. Finally, the phased array delay parameters derived from the numerical simulations are used to drive the custom transducer for transcranial precise focusing on phantom experiments [45,46]. In addition, the ultrasound field distribution is simulated in both pure water and uniform skull media. The uniform skull is modeled as a uniform rectangular block with a thickness of 7 mm, using the acoustic parameters of the skull, with a sound speed of 2900 m/s, density of 1912 kg/m^3^, and a sound absorption coefficient of 13 dB/cm/MHz. The acoustic parameters for the pure water medium are set as follows: sound speed of 1500 m/s, density of 997 kg/m^3^, and sound absorption coefficient of 1 dB/cm/MHz. Then, by fixing the position of the ultrasonic phased array, we simulated the transcranial ultrasound field distribution at different focal depths and focal spot offsets. All numerical simulations in this study are carried out using MATLAB. The computing hardware configuration is as follows: Intel(R) Core(TM) i7-13700K (3.40 GHz), 32 GB DDR4 and an NVIDIA GeForce RTX 3060 GPU (12 GB VRAM). Numerical simulations of the intact skull require approximately 20 min. However, when only a skull segment is adopted, the simulation time is reduced to 2 min.

### 2.2. Acoustoelectric Brain Imaging

First reported in 1946, the acoustoelectric (AE) effect is a fundamental physical phenomenon referring to the local variation in resistivity induced by the propagation of ultrasound through a medium, which subsequently leads to voltage changes [47]. Under focused ultrasound, the variation in resistivity satisfies the following relation:

(14) $$\Delta \rho =K\Delta P\rho_{0}$$

where ΔP is the ultrasound pressure change, Δρ is resistivity change, $\rho_{0}$ is the initial resistivity and K is the AE interaction coefficient, whose value is dependent on the medium property.

When the lead comprises a set of a pair of fixed electrodes, its sensitivity distribution is referred to as a “lead field” [48,49]. According to the reciprocity theorem, when a unit current is injected into the electrodes, a fabricated field is formed, and the current field distribution is identical to that of the lead field. Therefore, the voltage V across the lead can be calculated by integrating the dot product of the lead field current density ${\vec{J}\;}_{L}$ and the current source density ${\vec{J}}_{I}$. This formula can be expressed as

(15) $$V=∭\rho \;{\vec{J}}_{I}\cdot {\vec{J}}_{L}dxdydz$$

The key to tABI theory is the coupling of ultrasound field and brain electric fields. Based on the AE effect, the brain tissue resistivity ρ in the focal spot is equal to the sum of the initial resistivity and the resistivity change:

(16) $$\rho =\rho_{0}+\Delta \rho =\rho_{0}+K\rho_{0}\Delta P$$

According to the AE effect and lead field theory, the tABI recording voltage V  can be described as

(17) $$V=V_{LF}+V_{AE}=∭\left({\vec{J}}_{L}\cdot {\vec{J}}_{I}\right)\rho_{0}dx\;dy\;dz+∭({\vec{J}}_{L}\cdot {\vec{J}}_{I})(-K\rho_{0}\Delta P)dx\;dy\;dz$$

where $V_{LF}$ is the low-frequency signal which represents the brain electrical signal. $V_{AE}$ is the high frequency which represents the generated AE signal in the focal region of ultrasound. It is important to note that the AE signal exhibits distinct ultrasonic characteristics, with its frequency aligning with the pulse repetition frequency (PRF) of ultrasound, and it is significantly higher than the brain electrical signal.

(18) $$V_{AE}=∭({\vec{J}}_{L}\cdot {\vec{J}}_{I})(-K\rho_{0}\Delta P)d\Omega$$

where dΩ represents the focal spot of the ultrasound, typically on the millimeter scale, indicating that the AE signal contains high spatial location information. As described in the formula, the $V_{AE}$ signal also contains information about the source current density ${\vec{J}}_{I}$. Therefore, tABI can accurately detect brain electrical signals with high spatiotemporal resolution, thereby enabling the mapping of brain activity distributions through systematic brain scanning.

## 3. Materials and Methods

### 3.1. Transcranial Focused Experiment Method

In this study, a transcranial ultrasound field measurement platform is constructed, as shown in Figure 2. High-precision fixtures are used to fix the custom ultrasonic phased array and the skull. The ultrasonic phased array is positioned 3 mm below the skull, consistent with the numerical simulation setup. The phased array delay parameters derived from the numerical simulation are used to drive the custom ultrasonic phased array, with a single element excitation voltage of 90 V. Ultrasound pressure is measured using a needle hydrophone (NH1000, PA, Precision Acoustics Ltd. Hampton Farm Business Park, Higher Bockhampton, Dorchester, UK). The hydrophone is fixed on a three-dimensional motion device (C-scan), and the entire transcranial ultrasound field distribution is measured by moving the hydrophone. Ultrasound field data is collected and stored using the C-scan host. During measurements, degassed water is injected into the water tank to submerge the skull. The C-scan length is 8 mm in the X/Y direction and 12 or 15 mm in the Z direction, with a scanning step of 0.1 mm.

The phantom experiments provide measurement validation for all numerical simulation, including the ultrasound field in pure water, the transcranial ultrasound field of the phased array delay parameters derived from numerical simulation of pure water medium, the transcranial ultrasound field of the phased array delay parameters derived from numerical simulation homogeneous skull medium, and the transcranial ultrasound field of the phased array delay parameters derived from numerical simulation of the 3D skull model simulation. It is noteworthy that in all three conditions, the ultrasound focal length is set to 25 mm, specifically 15 mm below the skull, with the focal spot coordinates set at (0, 0, 25). In addition, the phantom experiments also measured the transcranial ultrasound field distribution at focal lengths of 22, 25, and 28 mm, as well as the transcranial ultrasound field distributions at focal spot coordinates of (0, 3, 25), (0, −3, 25), (3, 0, 25), and (−3, 0, 25).

### 3.2. tABI Experimental Methods

The tABI experimental setup is shown in Figure 2. The placement positions of the skull and the ultrasonic phased array remain unchanged. The hydrophone is removed and replaced with a custom-designed chamber, the bottom of which is covered with an ultrasonic window. The chamber is filled with 0.9% NaCl to simulate brain tissue, and current is injected through electrodes to simulate brain activity. 0.9% NaCl demonstrates considerable similarity to brain tissue in acoustic and electrical properties with brain tissue and features facile preparation and immediate availability, making it widely employed in AE effect experiments. The entire chamber structure is designed using SolidWorks software 2021 (Dassault Systemes SolidWorks Corp., Waltham, MA, USA) and fabricated using 3D printing technology (Shenzhen Future Workshop Technology Co., Ltd., Shenzhen, China), as shown in Figure 2c. The chamber has a rectangular structure, with dimensions of 13 mm in height, 35 mm in length, 24 mm in width, and a wall thickness of 2 mm. As shown in Figure 2c, three platinum-wire electrodes are placed inside the chamber: the activation source electrode (2, 1.5, 25), the recording electrode (2, 4.5, 25), and the reference electrode (17.88, 1.5, 25). Each electrode is inserted through holes in the chamber surface ring and adjusted along the depth direction to precisely align with the focused ultrasound beam. The source and recording electrodes are adjusted to the same depth to form a plane. A signal generator injects a sine wave signal through the activation source electrode into the chamber, with frequencies of 8/11/15 Hz and an amplitude of ±90 mV, generating a dipole source current field within the 0.9% NaCl solution chamber. All electrodes are connected to an amplifier panel (SynAmpsRT, 64 channels, NeuroScan, Charlotte, NC, USA). The signals are sampled at a frequency of 20 kHz.

During the tABI experiment, the ultrasonic phased array is driven to focus based on the delay parameters obtained from the numerical simulation of the 3D skull model. The pulse repetition frequencies (PRFs) of the ultrasound phased array are set to 968/1300/1700 Hz, with the focal length set to 25 mm. The ultrasonic phased array performs xy-plane scanning of the region, including the activation source, to capture the AE signals from the scanned area. Figure 2e illustrates a schematic of the ultrasound scanning plane, with a scanning step size of 2 mm. Along the x/y directions, the transducer scans 8 mm (x/y = −4 mm to 4 mm), resulting in a total of 25 focal spots. The measured signals consist of high-frequency AE signals and low-frequency activation source signals. The low-frequency signals can be removed using a band-pass filter with a PRF of ±15 Hz. The envelope of the high-frequency signals is averaged to obtain the measured value of the AE signals at the focal spots.

## 4. Results

### 4.1. Precise Focusing with Transcranial Ultrasound

#### 4.1.1. Transcranial Focused with the Conventional Model

**Figure 3:**
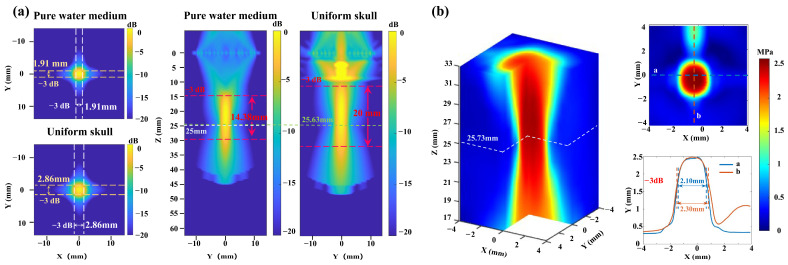
(**a**) The ultrasound field distribution in the pure water medium and the uniformly structured skull medium with numerical simulation experiment. “Uniform skull” indicates a uniform skull structure medium. (**b**) The ultrasound field distribution in the pure water medium with a phantom experiment.

In numerical simulation experiments, focusing of the ultrasonic phased array is achieved in both the pure water medium and the uniformly structured skull (thickness: 7 mm). The focal length of the ultrasound phased array is set to 25 mm. As shown in Figure 3a, the numerical simulation results indicate that the focal length of the ultrasound in pure water medium is 25 mm. Based on the −3 dB peak attenuation, the focal spot has a jujube pit shape, with a diameter of 1.91 mm in the xy-plane and a length of 14.38 mm along the z-axis in the pure water medium. When the ultrasound passes through the uniformly structured skull, the ultrasound focal length is 25.63 mm with a focus error of less than 0.63 mm. Based on the −3 dB peak attenuation, the focal spot has a jujube pit shape, with a diameter of 2.86 mm in the xy-plane and a length of 20 mm along the z-axis. The numerical simulation experiment results indicate that the ultrasonic phased array can achieve precise focusing, regardless of whether it is in the pure water medium or through a uniformly structured skull. But, after passing through the uniformly structured skull, the ultrasound focal spot size increases by 68%. Then, the delay parameters of the ultrasonic phased array in pure water medium, obtained through numerical simulation, are applied in phantom experiments. Hydrophone measurements are taken to assess the ultrasound field distribution of the ultrasound phased array in pure water. As shown in Figure 3b, the ultrasound focal length is 25.73 mm measured in pure water by the hydrophone with a 2.92% (0.73 mm) error between the phantom and simulation results, and the diameters of the focal spot are 2.10 mm and 2.30 mm in the xy-plane.

**Figure 4:**
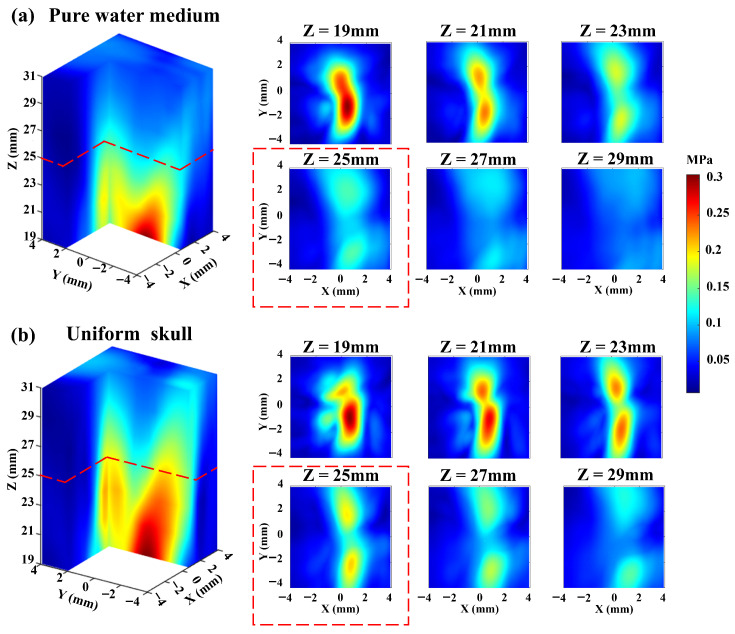
(**a**) The transcranial ultrasound field distribution generated by using the delay parameters derived from the pure water simulation experiment. (**b**) The transcranial ultrasound field distribution generated by using the delay parameters derived from the uniformly structured skull simulation experiment.

Then, the delay parameters derived from the pure water simulation experiment are applied to drive the ultrasonic phased array in the transcranial phantom experiment. As shown in Figure 4a, the ultrasound beam does not focus on the set focal spot, and the entire transcranial ultrasound field exhibits a divergent state. Only when Z = 19 mm does the ultrasonic wave achieve focusing in the xy-plane. Due to the limitation of the actual skull bowl structure, it is not possible to measure the ultrasound focal length. But the experimental results indicate that the ultrasound focal spot is less than 6 mm below the skull. Compared to the target set 15 mm below the skull, the focus error exceeds 40%. Experimental results indicate that the transcranial ultrasound field is significantly distorted and cannot be effectively guided by the ultrasound field of the pure water medium.

Furthermore, the delay parameters derived from the uniformly structured skull simulation experiment are also applied to drive the ultrasonic phased array in the transcranial phantom experiment. The results are shown in Figure 4b; although there is an increase in ultrasound energy and an enhanced focusing trend at the target location compared to the pure water medium delay parameters, the ultrasound field at the target remains divergent. The ultrasound focal spot is also less than 6 mm below the skull, and the ultrasound focus error exceeds 40%. This is attributed to the complex internal structure of the skull, with varying density and sound speed characteristics. Therefore, the shape and inhomogeneity of the skull model significantly affect the precision of focus. These results indicate that numerical simulations of the uniform skull and pure water models cannot enable transcranial ultrasound to be accurately focused at the target position during phantom experiments. Since precise focus of transcranial ultrasound is a fundamental prerequisite for tABI to accurately detect intracranial signals, conventional transcranial focusing models cannot support the application of tABI in intracranial electrical signal detection.

#### 4.1.2. Transcranial Focused with the Proposed Simulation Platform

**Figure 5:**
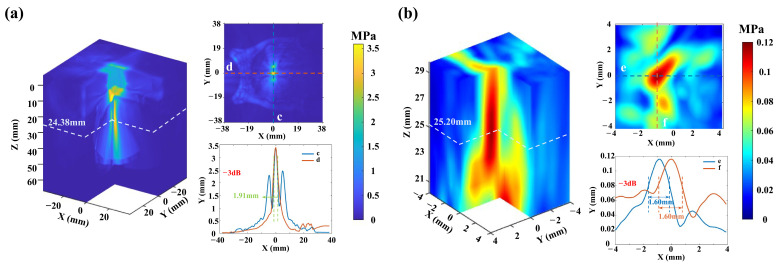
(**a**) The transcranial ultrasound field distribution in numerical simulation experiment. (**b**) The transcranial ultrasound field distribution in numerical phantom experiment.

The precise focusing simulation platform, combining a high-precision 3D skull structural model and a mathematical model, is used for transcranial ultrasound in numerical simulation and in phantom experiments. The numerical simulation results are shown in Figure 5a; ultrasound precisely focuses on the target region, with a focal length of 24.38 mm and a focus error of 2.48% (0.62 mm) relative to the target position. Based on the −3 dB peak attenuation, the focused spot has a jujube pit shape, with a diameter of 1.91 mm in the xy-plane. Then, the delay parameters derived from the high-precision 3D skull model simulation experiment are applied to drive the ultrasonic phased array in the transcranial phantom experiment. As shown in Figure 5b, the phantom experimental results are consistent with the numerical simulation, with a focal length of 25.20 mm, which deviates by 0.82 mm from the numerical simulation focal length. However, the focal spot coordinates measured by the hydrophone in the phantom experiment are (−0.74, 0.11, 25.20), showing a displacement of 0.74 mm along the x-axis, 0.11 mm along the y-axis, and 0.20 mm along the z-axis compared to the target coordinates. Based on the −3 dB peak attenuation, the focal spot is elliptic with a diameter of 1.91 mm in the xy-plane. The ultrasound pressure at the focal spot is 0.12 MPa, representing a 95% attenuation of the transcranial ultrasound pressure compared to the focal spot ultrasound pressure of 2.51 MPa in pure water medium (as shown in Figure 3b). These findings demonstrate that the proposed precise focusing simulation platform achieves precise transcranial ultrasound focusing with a focus error of 0.8%, in contrast to the uniform skull and pure water models, which fail to produce accurate focusing in phantom experiments. Therefore, the precise focusing simulation platform can effectively guide tABI to accurately detect and localize intracranial activation sources.

**Figure 6:**
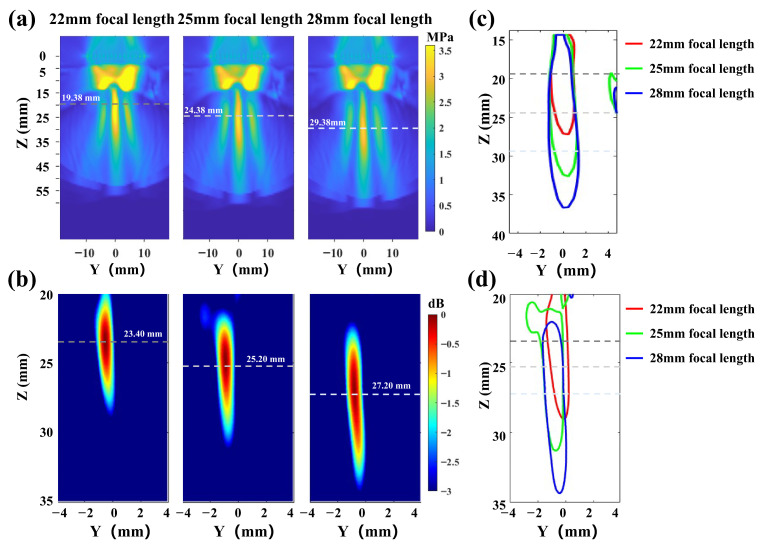
(**a**,**c**) The longitudinal focal spot distributions of transcranial ultrasound with different focal lengths—22 mm, 25 mm and 28 mm—in numerical simulation experiments. (**b**,**d**) The longitudinal focal spot distributions of transcranial ultrasound with different focal lengths—22 mm, 25 mm and 28 mm—in phantom experiment.

**Figure 7:**
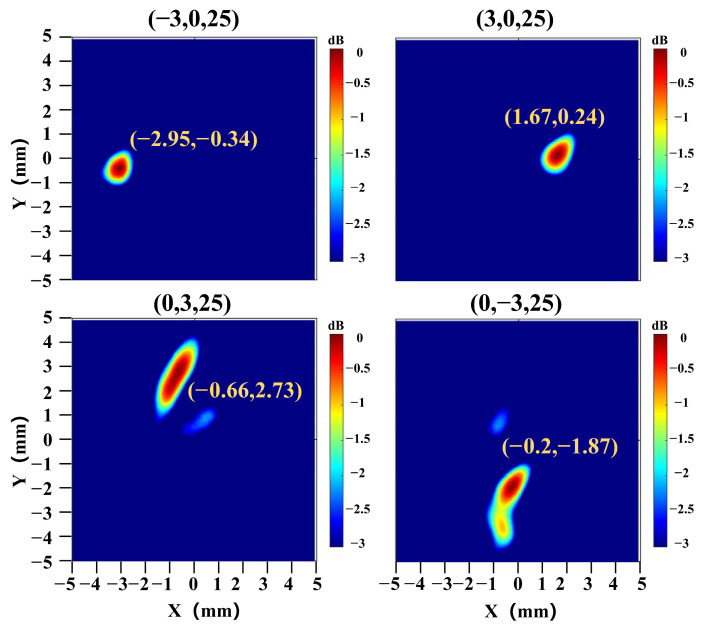
The transcranial ultrasound field distributions at different focal spots, (−3, 0, 25), (3, 0, 25), (0, −3, 25), (0, 3, 25), in the phantom experiment, when the focal length is fixed at 25 mm.

In addition, the transcranial ultrasound focusing results at different focal spots are presented in Figure 6 and Figure 7. The ultrasound focal lengths are set to 22 mm, 25 mm, and 28 mm, with the focal spots 12 mm, 15 mm, and 18 mm beneath the skull, respectively. As shown in Figure 6a, the actual focal lengths are 19.38 mm, 24.38 mm, and 29.38 mm, with a maximal error of 11.90% from the set positions in numerical simulation. As shown in Figure 6b, the actual focal lengths are 23.40 mm, 25.20 mm, and 27.20 mm, with a maximal error of 6.30% from the set positions in the phantom experiment. The results for different focal spots at the same focal length are shown in Figure 7, where the ultrasound focal length is set to 25 mm, and the focal spot coordinates are set to (−3, 0, 25), (3, 0, 25), (0, −3, 25), and (0, 3, 25). The actual focal spot coordinates are (−2.95, −0.34, 25), (1.67, 0.24, 25), (−0.20, −1.87, 25), and (−0.66, 2.73, 25). The actual focal spots match the set positions with a maximal error of 1.33 mm. These results demonstrate that the proposed simulation platform enables accurate intracranial focusing and scanning, thereby effectively guiding tABI to precisely detect brain activation sources with high spatiotemporal resolution.

### 4.2. tABI Precise Activation Source Localization Based on the Focusing Simulation Platform

**Figure 8:**
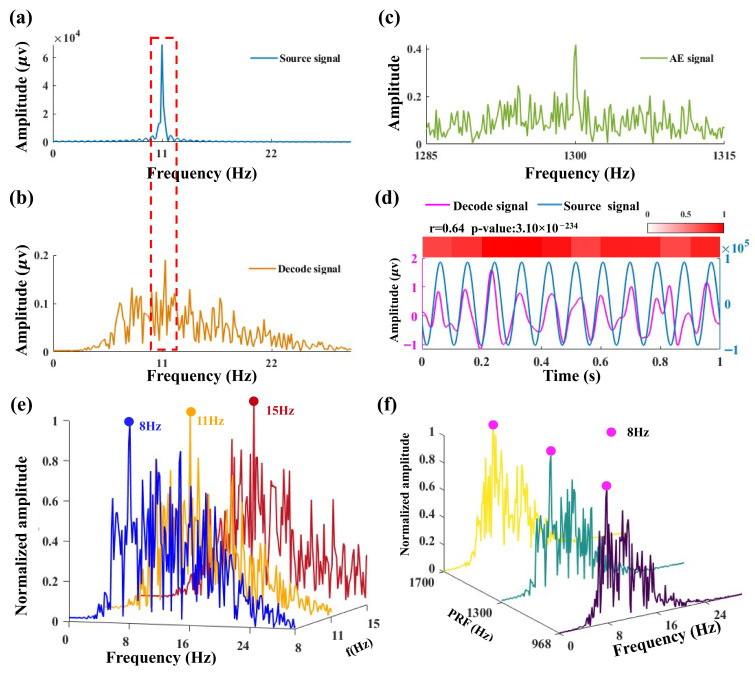
(**a**) The response of the source signal frequency. (**b**) The response of decoded AE signal frequency. (**c**) The response of AE signal frequency. (**d**) The correlation analysis of the decode signal and source signal. The correlation coefficient image uses a sliding window width of 0.1 s. (**e**) The response of decoded AE signal frequency with various source signal frequencies (8, 11, and 15 Hz), when the ultrasound PRF is all 1300 Hz. (**f**) The response of decoded AE signal frequency when the PRFs are set to 968, 1300, and 1700 Hz, but source signals are all 8 Hz.

As shown in Figure 8a, when the signal generator injects a sine wave signal with a frequency of 11 Hz and an amplitude of ±90 mV into the chamber through the source electrode, the low-frequency signal recorded by the recording electrode exhibits a significant frequency-amplitude response at the 11 Hz frequency. The ultrasonic phased array is focused on the source electrode position, with the PRF set to 1300 Hz, to perform ultrasound encoding on the injected sine wave signal. The AE signal is extracted via high-frequency band filtering (1285–1315 Hz) applied to the measured signal. As shown in Figure 8c, the AE signal exhibits a significant frequency-amplitude response at 1300 Hz, consistent with the ultrasound PRF. Then, the decoded AE signal is obtained by decoding the AE signal, and it is consistent with the measured signal. As shown in Figure 8b, the decoded AE signal demonstrates a significant frequency-amplitude response at 11 Hz. To further evaluate the performance of the tABI technique, the time domain consistency between the decoded AE signal and the source signal is verified. As shown in Figure 8d, both the decoded AE signal period and the source signal period are 91 ms, and their time waveforms display a strong linear correlation (Pearson correlation coefficient r = 0.64, within a 1 s time period, *p* < 0.001). In addition, a control experiment without ultrasound is designed, and the results are shown in Supplementary Figure S1. When a sine wave signal with a frequency of 8 Hz and an amplitude of ±90 mV is injected into the chamber through the source electrode, the low-frequency signal recorded exhibits an obvious frequency-amplitude response at 8 Hz. However, when the high-frequency signal is extracted by high-frequency band filtering (1285–1315 Hz) applied to the measured signal, no significant frequency-amplitude response is observed at 1300 Hz. Furthermore, after decoding the high-frequency signal, no obvious frequency-amplitude response appears at 8 Hz, and the time-domain waveforms showed no corresponding correlation.

Then, the frequency response of the decoded AE signal under different PRF or different source signal frequencies was investigated. In Figure 8e, tABI experiments are conducted with a fixed PRF of 1300 Hz, and sine wave signals with frequencies of 8 Hz, 11 Hz, and 15 Hz are injected into the chamber, respectively. The frequency spectrum of the decoded AE signal exhibits distinct peaks at 8 Hz, 10 Hz, and 11 Hz. Additionally, as shown in Figure 8f, the frequency spectrum of the decoded AE signal is obtained with ultrasound PRFs set to 968 Hz, 1300 Hz, and 1700 Hz, respectively, while the injected source signal is a sine wave at 8 Hz. In each case, the decoded AE signal exhibited a strong amplitude response at 8 Hz. The results indicate that tABI can perform ultrasound encoding and decoding at different repetition frequencies, accurately detecting source signals with various frequencies.

**Figure 9:**
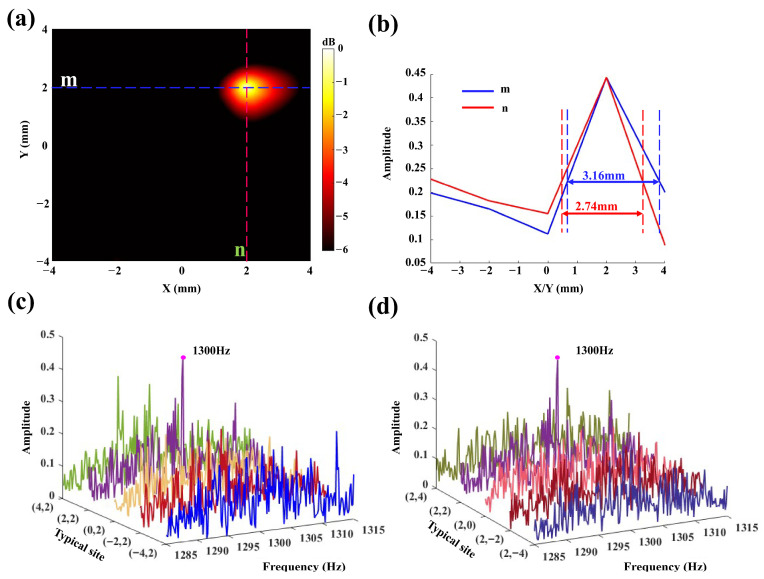
(**a**) The activation source location by tABI. (**b**) The amplitude distribution along the marked lines m and n of (**a**). (**c**,**d**) The frequency responses of typical sites that include points (4, 2), (2, 2), (0, 2), (−2, 2), (−4, 2), (2, 4), (2, 0), (2, −2), (2, −4), in marked lines m and n of (**a**).

Finally, we used the tABI technique to map the activation source distribution within the pig skull. As shown in Figure 9a, the activation source map indicates that the source is located at coordinates (2,2), which aligns with the actual location of the activation source at coordinates (2,1.50). The results demonstrate that the tABI technique can accurately localize the intracranial activation source, with an error of 0.50 mm. A quantitative analysis of the activation source imaging results is performed. At the location of the activation source, a line m (blue dashed line, y = 2) is drawn along the x-axis, and a line n (red dashed line, x = 2) is drawn along the y-axis, as shown in Figure 9a. The frequency amplitude distribution is plotted along both lines m and n. As shown in Figure 9b, along the y-axis direction (line n), the −3 dB peak width is 2.74 mm. Similarly, along the x-axis direction (line m), the diameter of the activation source region is 3.16 mm. Nine typical ultrasound encoding positions are selected along lines m and n to plot the frequency distribution of the AE signals. As shown in Figure 9c,d, a significant amplitude response at 1300 Hz is observed only at the ultrasound encoding position (2,2). The results indicate that the spatial resolution achieved by tABI is at least 3.16 mm, confirming that tABI can localize intracranial activation sources with millimeter-scale precision.

## 5. Discussion

In this paper, we focus on precise transcranial ultrasound focusing through numerical simulation, thereby enhancing tABI’s ability to detect and localize intracranial electrical signals. However, due to the complexity and heterogeneity of the skull structure, the ultrasound delay parameters derived from simulations in pure water and uniform skull media cannot guide the actual transcranial ultrasound, leading to a divergent ultrasound pattern in the target region. The precision focusing simulation platform proposed enables accurate transcranial ultrasound focusing, which plays a crucial role in guiding tABI for the precise detection of intracranial electrical signals. The results from phantom experiments indicate that the ultrasonic phased array can precisely focus on the target location within the pig skull, with capabilities for different depths and focused scans, yielding a maximum focus error of 1.33 mm and a minimum error of 0.05 mm. By comparison, Marquet et al. reported a localization error of 0.70 mm in in vitro experiments on monkey and human skull samples, also employing time delays calculated from numerical simulation. Based on the precision focusing of transcranial ultrasound, we achieved accurate detection and source localization of intracranial electrical signals using tABI, with an activation source localization error of 0.50 mm and a decoding accuracy exceeding 0.64.

This study also has some limitations and still needs to be further improved. First, this paper focuses primarily on the distortion and displacement of transcranial ultrasound, without addressing the prediction and compensation of ultrasound pressure attenuation. Experimental results indicate that, compared to the pressure at the focus in pure water media, the pressure after transcranial passage is attenuated by 95%. In subsequent studies, we will further construct a 3D skull model based on more accurate acoustic parameters and skull structure, incorporating a pressure compensation algorithm to address the transcranial ultrasound pressure attenuation issue. Furthermore, based on the discharge frequencies of alpha and beta rhythms in EEG, we will investigate the AE signal and decoded AE signal responses of the intracranial activation source at 8 Hz, 11 Hz, and 15 Hz. However, the source signals are modeled as sinusoidal waves with amplitudes set at ±90 mV, which exceed the typical waveform and amplitude of actual EEG signals. Further studies are therefore required to investigate the performance of EEG signal detection and source localization at lower, physiologically realistic amplitudes. Finally, in the tABI experiments, we only consider the skull as the influencing factor, which still presents a significant gap preventing us from achieving non-invasive, high-spatiotemporal-resolution neural detection technology. In future work, we will consider the influence of multi-layered biological tissues in the head, such as the scalp, skull, cerebrospinal fluid, and brain tissue, on transcranial precision focusing and AE signal responses, further advancing tABI towards clinical diagnostic and therapeutic applications [50].

## 6. Conclusions

We propose a method that combines time-reversal phase control with high-precision 3D skull model numerical simulations for ultrasonic phased array precision focusing. Based on transcranial precision focusing, we achieved accurate detection of intracranial electrical signals and precise localization of activation source positions using tABI. The depth of precision focusing can reach up to 18 mm within the skull, with an error of less than 2 mm, while the activation source localization error using tABI is less than 0.50 mm. This result lays a solid foundation for the future use of tABI in non-invasive, high-spatiotemporal-resolution mapping of deep brain activity distributions. It contributes to the non-invasive and efficient clinical diagnosis and research of brain diseases, such as epilepsy, Parkinson’s disease, and Alzheimer’s disease.

## Data Availability

The data presented in this study are available on request from the corresponding author.

## Supplementary Materials

The following supporting information can be downloaded at: https://www.mdpi.com/article/10.3390/s26092715/s1. Supplementary Figure S1: (a) The response of the source signal frequency. (b) The response of decoded signal frequency. (c) The response of high frequency signal frequency with. (d) The correlation analysis of decode signal and source signal. The correlation coefficient image uses a sliding window width of 0.1 s.
