# Supplementary material for: A Precise Focusing Simulation Platform for Transcranial Acoustoelectric Brain Imaging
Source: Sensors (Basel). 2026 Apr 28;26(9):2715. doi: 10.3390/s26092715 (PMC13166024; doi:10.3390/s26092715)
Supplement: Supplementary file 1 [file sensors-26-02715-s001.zip › sensors-4234943-supplementary.pdf]

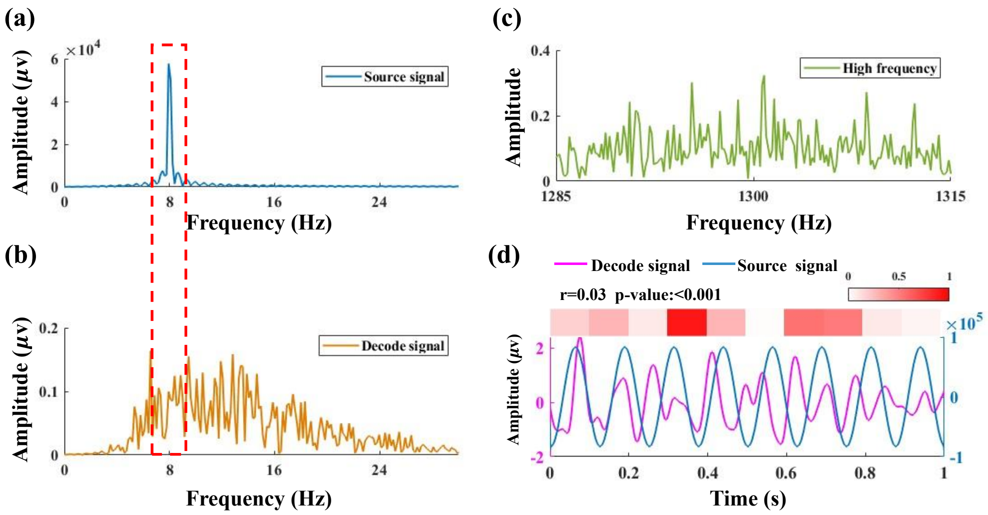

Supplementary Figure S1. (a) The response of the source signal frequency. (b) The response of decoded signal frequency. (c) The response of high frequency signal frequency with. (d) The correlation analysis of decode signal and source signal. The correlation coefficient image uses a sliding window width of 0.1s.
